# Supplementary material for: Single-Round LDCT Screening in Men Aged ≥ 70 Years: Prevalence of Pulmonary Nodules and Lung Cancer Detection
Source: Cancers (Basel). 2025 Jul 11;17(14):2318. doi: 10.3390/cancers17142318 (PMC12293317; doi:10.3390/cancers17142318)

Table S1. Baseline Characteristics of Study Participants Compared with Previous Lung Cancer Screening Populations

Characteristic	This study	NLST (Church et al., 2013)[1]	NELSON (Horeweg et al., 2014)[2]	UKLS (Field et al., 2021)[3]
**Total participants of taking LDCT(N)**	1,409	26,309	7,155	4,055
**Sex (% male)**	100%	59%	84%	75%
**Age group distribution (%)**	70–74:68.1%<br>75–79:16.7%<br>80–84: 9.2%<br>85–89:5.4%<br>≥90: 0.6%	55-59:42.7%<br>60-64:30.6%<br>65-6917.8%<br>70-74:8.8%	50-59 :2%<br>60-69 64%<br>70-76:34%	Not detailed
**COPD prevalence (%)**	41.7%	Not specifically reported	Not specifically reported	History of respiratory disease:52%
**Smoking status (%)**	Current smoker: 31.9<br>Former smoker: 60.7<br>Never smoker: 7.5	Current smoker: 48.1<br>Formal smoker: 51.9	Current smoker: 55<br>Formal smoker: 45	Current smoker 38<br>Formal smoker 62
**Median pack-years (smokers)**	40 [IQR: 25–50]	Not specifically reported	40 [IQR: 29.7–49.5]	Not specifically reported

Table S2. Comparative Characteristics of Malignant vs. Non-Malignant Screen-Detected Lung Nodules

Characteristics	Total nodule (N = 786)	Lung Cancer (n=31)	Non-Cancerous (n=755)	*P*-value
**Nodule size (mm)**	6.6 ±6.2	25.0 ± 17.9	5.8± 3.6	<0.001
**Nodule type, no. (%)**				0.001
**Solid**	637 (81.0)	22 (71.0)	615 (81.5)	
**Partial solid**	65 (8.3)	8 (25.8)	57 (7.6)	
**Ground-glass opacity**	84 (10.7)	1 (3.2)	83 (11.0)	
**Nodules (%)**				<0.001
**Single**	408 (51.9)	26 (83.9)	382 (50.6)	
**Multiple**	378 (48.1)	5 (16.1)	373 (49.4)	
**Lung-RADS score**				<0.001
**2**	607 (77.2)	5 (16.1)	602 (79.7)	
**3**	95 (12.1)	2 (6.5)	93 (12.3)	
**4A**	55 (7.0)	9 (29.0)	46 (6.1)	
**4B**	22 (2.8)	8 (25.8)	14 (1.9)	
**4X**	7 (0.9)	7 (22.6)	0 (0.0)	
**Follow-up outcome**				
**Disappeared**	69 (8.8)	0 (0.0)	69 (9.1)	0.101
**Size decrease**	84 (10.7)	0 (0.0)	84 (11.1)	0.129
**Size increase**	50 (6.3)	13 (41.9)*	37 (4.9)	<0.001

*Other Eighteen patients were diagnosed with lung cancer during the initial diagnostic work-up and did not receive subsequent follow-up LDCT

Table S3. Comparison of lung nodule detection rates between the current study and the ImaLife cohort

**Age groups**	**Current study**		**ImaLife cohort (male participants)[4]**	
	Nodules ≥4mm	Nodules ≥6mm	Nodules ≥30 mm^3	Nodules≥100 mm^3
**70–74**	56.1	20.3	56.1	17.3
**75–79**	54.5	26.6	58.5	22.3
**80 +**	55.8	30.0	60.7	24.4

(1) Church, T. R.; Black, W. C.; Aberle, D. R.; Berg, C. D.; Clingan, K. L.; Duan, F.; Fagerstrom, R. M.; Gareen, I. F.; Gierada, D. S.; Jones, G. C.; et al. Results of initial low-dose computed tomographic screening for lung cancer. *N Engl J Med* **2013**, *368* (21), 1980-1991. DOI: 10.1056/NEJMoa1209120 From NLM.
(2) Horeweg, N.; Scholten, E. T.; de Jong, P. A.; van der Aalst, C. M.; Weenink, C.; Lammers, J. W.; Nackaerts, K.; Vliegenthart, R.; ten Haaf, K.; Yousaf-Khan, U. A.; et al. Detection of lung cancer through low-dose CT screening (NELSON): a prespecified analysis of screening test performance and interval cancers. *Lancet Oncol* **2014**, *15* (12), 1342-1350. DOI: 10.1016/s1470-2045(14)70387-0 From NLM.
(3) Field, J. K.; Vulkan, D.; Davies, M. P. A.; Baldwin, D. R.; Brain, K. E.; Devaraj, A.; Eisen, T.; Gosney, J.; Green, B. A.; Holemans, J. A.; et al. Lung cancer mortality reduction by LDCT screening: UKLS randomised trial results and international meta-analysis. *The Lancet Regional Health – Europe* **2021**, *10*. DOI: 10.1016/j.lanepe.2021.100179 (accessed 2025/06/06).
(4) Cai, J.; Vonder, M.; Pelgrim, G. J.; Rook, M.; Kramer, G.; Groen, H. J. M.; de Bock, G. H.; Vliegenthart, R. Distribution of Solid Lung Nodules Presence and Size by Age and Sex in a Northern European Nonsmoking Population. *Radiology* **2024**, *312* (2), e231436. DOI: 10.1148/radiol.231436 From NLM.

Figure S1. Baseline Characteristics of Study Participants Compared with Previous Lung Cancer Screening Populations

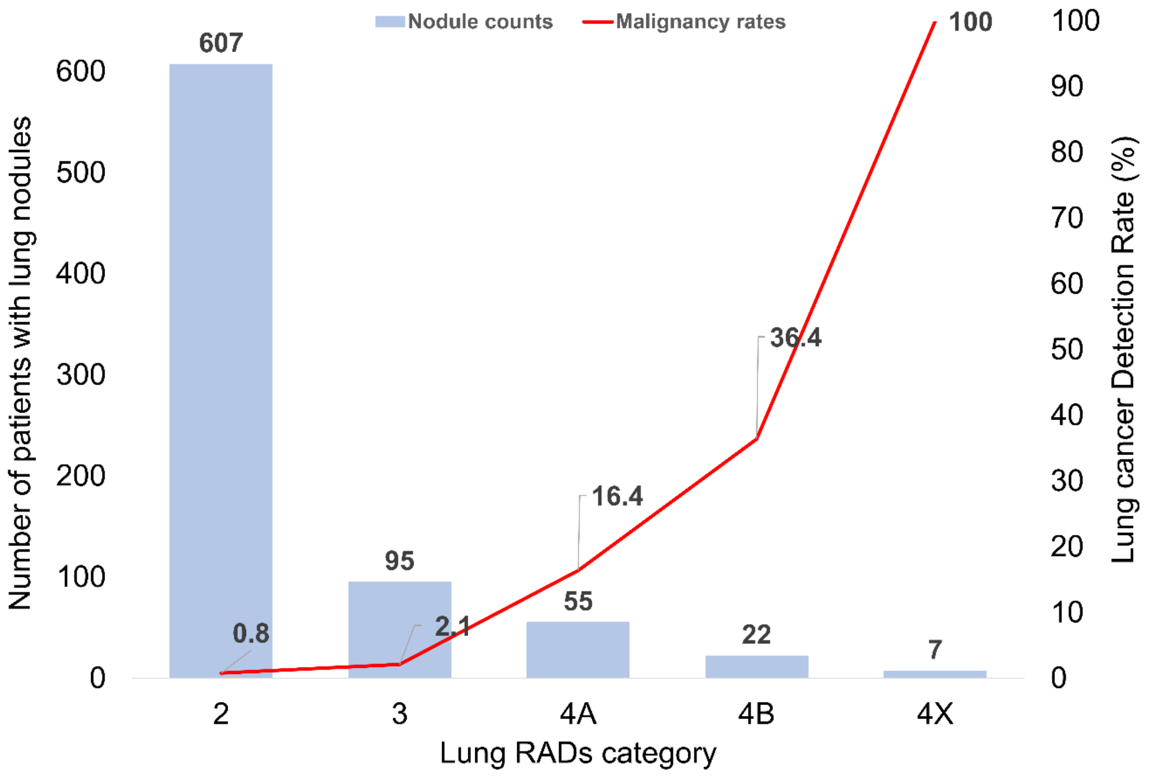

Supplement: Supplementary file 1 [file cancers-17-02318-s001.zip › cancers-3648557-supplementary_new.pdf]
